# Epigenetic regulation of mycorrhizal symbioses: from plastic responses to transgenerational legacies

**DOI:** 10.1111/nph.70982

**Published:** 2026-02-06

**Authors:** Gerson Beltrán‐Torres, Henry J. De La Cruz, Stéphane Maury, Martina Janoušková, Claire Veneault‐Fourrey, Vít Latzel, Pierre‐Emmanuel Courty, Harold Duruflé, Jörg Tost, Iris Sammarco

**Affiliations:** ^1^ Institute of Botany Czech Academy of Sciences Zámek 1 252 43 Průhonice Czechia; ^2^ Department of Botany, Faculty of Science Charles University Benátská 2 128 01 Prague Czechia; ^3^ Physiology, Ecology and Environment (P2e), USC1328 INRAE, UR1207 University Orléans 45067 Orléans France; ^4^ Université de Lorraine, INRAE Interactions Arbres‐Microorganismes (IAM) 54000 Nancy France; ^5^ Université Bourgogne Europe, Institut Agro Dijon, INRAE UMR Agroécologie 21000 Dijon France; ^6^ INRAE, ONF, BioForA, UMR 0588 45075 Orléans France; ^7^ Laboratory for Epigenetics and Environment Centre National de Recherche en Génomique Humaine, CEA‐Institut de Biologie François Jacob, Université Paris Saclay 2 Rue Gaston Crémieux 91000 Evry France

**Keywords:** adaptation and resilience, environmental change, epigenetic regulation and inheritance, mycorrhizal symbiosis, plant–microorganism interactions, reproductive modes, within‐ and across‐generational plasticity

## Abstract

Mycorrhizal symbioses represent one of the most widespread and ecologically significant plant–microbe interactions, shaping plant nutrition, stress resilience, and ecosystem functioning. Beyond their role in nutrient exchange and systemic defense, growing evidence suggests that these symbioses also influence plant plasticity within and across generations through epigenetic regulation. These mechanisms operate throughout the mutualistic interaction, from fungal recognition and root colonization to symbiosis functioning, by regulating gene networks that control signaling, defense suppression, and nutrient exchange. By integrating environmental cues into potentially heritable gene regulatory states, epigenetic regulation fine‐tunes within‐generation responses and may also contribute to effects across generations, thereby influencing adaptation and resilience. The extent of mycorrhiza‐induced epigenetic inheritance likely depends on the host's reproductive strategy and lifespan. Clonal propagation and shorter‐lived hosts tend to preserve epigenetic marks, whereas sexual reproduction and longer‐lived species show partial resetting. This contrast shapes offspring performance, ecological interactions, and evolutionary trajectories. Here, we synthesize current knowledge on the epigenetic regulation of mycorrhizal symbioses, draw parallels with other plant–microorganism interactions (including plant–pathogens and plant–endophytes), highlight its role in within‐generation plasticity and propose a potential role across generations. We outline future research directions to disentangle the stability, ecological relevance, and evolutionary significance of mycorrhiza‐mediated epigenetic inheritance.

**Table:** 

	Contents	
	Summary	1438
I.	Introduction	1439
II.	Epigenetic regulation of mycorrhizal symbioses	1441
III.	From within‐ to across‐generational epigenetics: knowledge gaps, challenges, and applications	1445
IV.	Conclusions	1448
	Acknowledgements	1448
	References	1448

## I. Introduction

Plants exist as holobionts, that is integrated, dynamic entities comprising the host and its associated microbiota (Margulis, 1993; Vandenkoornhuyse *et al*., 2015). This perspective has shaped our understanding of plant ecology and evolution, highlighting that phenotypic plasticity, stress resilience, and adaptive potential emerge from not only plant genomes but also interactions with associated microbiota (Zilber‐Rosenberg & Rosenberg, 2008).

The plant microbiota forms a diverse community of soil and phyllosphere microorganisms, ranging along a continuum from mutualists to antagonists; these microbes influence plant nutrient acquisition, growth, and defense (Richardson *et al*., 2009; Bulgarelli *et al*., 2013; Compant *et al*., 2019). Among them, mycorrhizal fungi are evolutionarily ancient root symbionts, associated with over 80% of terrestrial plant species, and play a central role in mediating plant–soil interactions (Cairney, 2000; Smith & Read, 2008; Tedersoo *et al*., 2020). Unlike many other root‐associated microorganisms, mycorrhizal fungi form specialized intracellular structures that mediate bidirectional exchange of carbon and nutrients, and trigger extensive reprogramming of plant development, defense, and metabolism at both local and systemic levels (Nehls *et al*., 2016; Bedini *et al*., 2018; Basso *et al*., 2020).

Mycorrhizal associations have evolved multiple times and include anatomically and functionally distinct types (Box 1) (Smith & Read, 2008; Brundrett & Tedersoo, 2018). These associations substantially enhance plant nutrient uptake, stress resilience, and competitive ability, thereby shaping plant communities and ecosystem functioning (Hoeksema *et al*., 2010; Bennett & Groten, 2022). Importantly, outcomes of mycorrhizal symbiosis shift along a mutualism–antagonism continuum depending on the plant and fungal genotypes, resource availability, and environmental conditions (Johnson *et al*., 1997; Hoeksema *et al*., 2010; Johnson, 2010), making this a powerful system to understand how plants regulate symbioses under fluctuating environments (Cavagnaro *et al*., 2021; Inoue *et al*., 2024). Although plants exert tight control over colonization and resource exchange through complex signaling networks (Nehls *et al*., 2016; Bedini *et al*., 2018; Wipf *et al*., 2019; Basso *et al*., 2020), the molecular mechanisms underlying context‐dependent plasticity remain poorly understood.

**Fig. 1:**
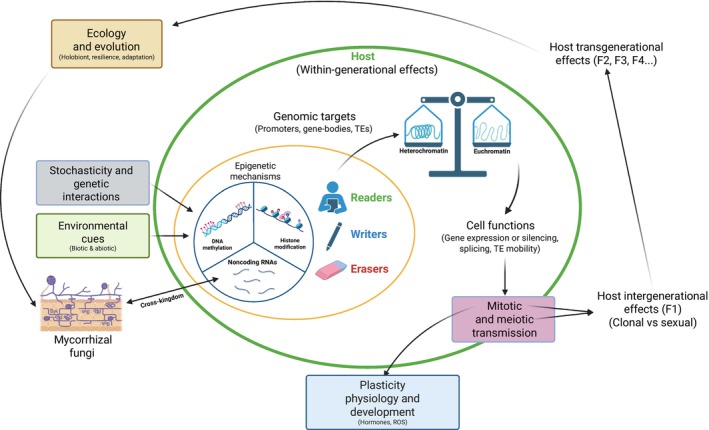
Epigenetic mechanisms linking mycorrhizal interactions and transgenerational responses. Epigenetic mechanisms are shaped by diverse inputs, including environmental cues (e.g. mycorrhizal symbioses, pathogens, drought, nutrient limitation), stochastic epimutations (random changes in epigenetic marks), and interactions with genetic variation. These factors act through ‘writers’, ‘erasers’, and ‘readers’, which establish or interpret the three main epigenetic mechanisms: DNA methylation, histone marks, and small RNAs (sRNAs). Cross‐kingdom sRNAs may mediate bidirectional (double‐headed arrow) communication between host and fungus, shaping epigenetic modifications in both partners. As indicated by the single‐headed arrow, the resulting epigenetic modifications balance chromatin states (euchromatin vs heterochromatin) at genomic targets such as gene promoters, gene bodies, and transposable elements (TEs). Through this dynamic balance, epigenetic regulation controls functional processes such as gene expression, alternative splicing, and TE activity, ultimately influencing plant and fungal physiology and development (e.g. hormonal pathways, ROS signaling, growth, and reproduction). Epigenetic information in the host can be transmitted mitotically (clonal memory) or meiotically (sexual inheritance). Depending on its stability, epigenetic variation can lead to intergenerational effects (first offspring generation, F1) or transgenerational effects (multiple offspring generations, F2, F3, F4, etc.). Together, these processes may shape the ecology and evolution of mycorrhizal interactions, influencing adaptive potential under global change. This figure was created in BioRender (BioRender.com/8rq6i2c).

Although direct evidence from mycorrhizal systems remains limited, epigenetic mechanisms provide a plausible system mediating plant–mycorrhizal interactions, particularly their context dependence (i.e. the variable outcomes of mycorrhizal associations across environmental conditions, partner identities, and plant physiological states). Epigenetics, defined as the heritable regulation of gene expression without changes in the underlying DNA sequence (Allis & Jenuwein, 2016), operates through multiple mechanisms, including DNA methylation, histone modifications, chromatin remodelers, and noncoding RNAs (ncRNAs), which act in concert to modulate genome accessibility and transcriptional activity (Boxes 2, 3) (Zhang *et al*., 2018; Alonso *et al*., 2019; Bewick *et al*., 2019). These mechanisms can integrate diverse inputs, including biotic and abiotic environmental cues, genetic background, and stochasticity (i.e. stochastic epimutations), into chromatin states that regulate gene expression (Fig. 1) (Angers *et al*., 2020; Biwer *et al*., 2020; Kramer *et al*., 2023; Zanetti *et al*., 2024). Although such processes are only beginning to be characterized in mycorrhizal systems (Box 2; Chaturvedi *et al*., 2021), epigenetic regulation in both plants and fungi operates through interconnected chromatin‐based layers. In plants, DNA methylation marks (Box 3) can be maintained through mitosis and, in some cases, through meiosis, especially in the CG and CHG methylation sequence contexts (where H= A, T or C), whereas CHH methylation is more dynamic and relies on continual *de novo* establishment (Law & Jacobsen, 2010). These patterns can also be reset through active or passive demethylation (Niederhuth & Schmitz, 2017; Gallego‐Bartolomé, 2020). Histone modifications (e.g. acetylation and methylation) and histone variants, together with chromatin remodelers, fine‐tune chromatin accessibility and establish transcriptional memory (Lämke & Bäurle, 2017). Small RNAs (sRNAs) link these processes by directing DNA methylation, recruiting chromatin modifiers, and even crossing kingdoms to mediate bidirectional plant–fungus communication (Matzke & Mosher, 2014; Wang *et al*., 2016). This cross‐kingdom transfer, well‐documented in pathogenic systems (Weiberg *et al*., 2013; Cai *et al*., 2018), has been shown to occur in ectomycorrhiza (EcM) (Wong‐Bajracharya *et al*., 2022) and arbuscular mycorrhizal fungi (AMF) (Silvestri *et al*., 2025). In addition, a protein effector from an AMF was shown to regulate host gene expression through an epigenetic mechanism (Wang *et al*., 2021), providing direct evidence for epigenetic influence during symbiosis.

Importantly, epigenetic modifications differ in stability: Some are transient, whereas others leave inheritance‐prone chromatin footprints that persist mitotically (‘clonal memory’) or across meiosis (‘sexual inheritance’). These differences underlie within‐generational, intergenerational, and transgenerational effects. Following prevailing terminology (Jablonka & Raz, 2009; Heard & Martienssen, 2014), *within‐generation effects* are restricted to an individual's lifetime, whereas *across‐generational effects* encompass both inter‐ and transgenerational influences. *Intergenerational effects* refer to parental influences that persist in the immediate progeny. In addition to inherited epigenetic states, these effects may arise from other mechanisms such as nutrient or hormonal provisioning (Herman & Sultan, 2011) or, in some symbiotic systems, the vertical transmission of microorganisms (e.g. endophytes; Afkhami & Rudgers, 2008). By contrast, *transgenerational effects* refer to heritable trait or regulatory changes that persist beyond direct parental exposure, into the second generation and beyond in sexual systems, thereby implying transmission through meiotic reprogramming (Paszkowski & Grossniklaus, 2011; Gundel *et al*., 2017; Cao & Chen, 2024).

Understanding how mycorrhizal fungi influence plant epigenetic processes is essential for revealing how biotic cues affect offspring performance, stress resilience, and adaptation. Reproductive mode may influence the potential for epigenetic inheritance: clonal and sexual reproduction differ in their capacity to maintain environmentally induced epigenetic modifications and in the ecological consequences of such retention (Verhoeven & Preite, 2014; Sammarco *et al*., 2025). Clonal propagation can preserve environmentally associated methylation patterns across vegetative generations (Díez Rodríguez *et al*., 2022; Sammarco *et al*., 2024; Vanden Broeck *et al*., 2024), whereas sexual reproduction partially resets DNA methylation marks during meiosis and fertilization (Wibowo *et al*., 2016). Stochastic epimutations also contribute to heritable variation and may accumulate more readily in clonal than in sexual lineages (van der Graaf *et al*., 2015; Wibowo *et al*., 2022).

From an ecological perspective, transgenerational epigenetic memory of mycorrhizal associations may play a less prominent role in clonal than in sexual offspring, potentially leading to different evolutionary implications across reproductive modes. Clonal offspring typically remain near the parental environment and share resources via vegetative connections (stolons or rhizomes), thereby buffering environmental heterogeneity and reducing dependence on mycorrhizal fungi for nutrient acquisition (Verhoeven *et al*., 2010; Latzel *et al*., 2025). By contrast, sexually produced offspring are often dispersed into novel environments (depending on species‐specific seed dispersal traits), increasing their reliance on establishing effective mycorrhizal associations (Latzel *et al*., 2025). It is therefore crucial to assess how these epigenetic and ecological dynamics interact to shape across‐generational effects.

Beyond reproductive mode, the temporal mismatch between lifespan and environmental predictability may also shape mycorrhiza‐induced epigenetic effects. In short‐lived herbaceous species (many AM hosts), generation times are brief, and offspring environments resemble parental conditions, potentially making mycorrhiza‐induced epigenetic signals adaptive (Varga & Soulsbury, 2019). Conversely, in long‐lived trees (typical EcM hosts), transgenerational epigenetic effects may be less informative for offspring success because sexual reproduction events can be separated from offspring germination by several decades (Vigneaud *et al*., 2023). By contrast, across‐generational effects, such as intergenerational influences that act directly on the next reproductive cycle, can still operate in these species. We therefore hypothesize that short‐lived AM hosts that reproduce clonally will be the most permissive systems for mycorrhiza‐induced epigenetic inheritance, combining rapid generational turnover with the capacity to preserve epigenetic marks across vegetative generations.

While epigenetic studies in AM and EcM interactions are gradually emerging, virtually no comparable data exist for ericoid or orchid mycorrhizas, which significantly limits our ability to generalize epigenetic patterns across mycorrhizal types.

Building on the mechanistic and inheritance framework outlined previously, this review synthesizes evidence for plant epigenetic regulation of (1) mycorrhiza establishment, including coordinated crosstalk between immune and hormone signaling pathways, and (2) plant–mycorrhizal nutrient exchange, particularly the symbiotic trade in which plants provide carbon to the fungus in return for nitrogen and phosphorus. For each process, we assess evidence for DNA methylation, histone modifications, and sRNA involvements during symbiosis while evaluating potential across‐generational outcomes. We conclude by highlighting key knowledge gaps, methodological challenges, and future applications, and by proposing experimental priorities aimed at directly connecting molecular epigenetic marks to ecological and evolutionary outcomes to determine when mycorrhiza‐induced epigenetic states contribute to plant adaptation and resilience (Richards *et al*., 2017; Stergachis *et al*., 2020; Dezem *et al*., 2024).

Box 1 **Main types of mycorrhizal symbioses**

Arbuscular mycorrhiza (AM) is the oldest and most widespread type, formed by obligate biotrophic fungi Glomeromycota (Spatafora *et al*., 2016). It involves *c*. 72% of vascular plants including most crops (Brundrett & Tedersoo, 2018), dominates grasslands and tropical forests, and is characterized by arbuscules penetrating plant cortical cells to enable nutrient exchange. Extraradical hyphae are aseptate, multinucleate, and lack saprotrophic capacity, making AM fungi fully dependent on their hosts. Establishment is orchestrated by the conserved common symbiosis signaling pathway (CSSP) (Radhakrishnan *et al*., 2020), which permits intracellular fungal growth and suppresses host immune response. AM is the most studied mycorrhizal association, providing a key framework for understanding mycorrhizal physiology.

Ectomycorrhiza (EcM) evolved independently in multiple Basidiomycetes and Ascomycetes lineages and predominates in long‐lived trees of boreal and temperate forests, where soils are nutrient‐poor but organic‐rich. In contrast to AM fungi, EcM fungi colonize only the root surface and apoplast, forming a mantle around roots and the intercellular Hartig net as the nutrient‐exchange interface. EcM establishment only partly relies on the CSSP (Garcia *et al*., 2015; Becquer *et al*., 2019) and exhibits distinct regulatory dynamics for nutrient exchange compared to AM (Tedersoo & Bahram, 2019).

Some trees can host both AM and EcM fungi, indicating complex interactions beyond single‐type colonization (Teste *et al*., 2020).

Two other widespread but less‐studied types are ericoid and orchid mycorrhizas. Ericoid mycorrhiza involves Ascomycetes colonizing epidermal cells of ericaceous plants in acidic, organic‐rich soils, forming dense intracellular coils with degradative abilities (Vohník, 2020). Orchid mycorrhiza involves Basidiomycetes colonizing cortical cells, forming pelotons that are later digested by the plant, supporting seed germination and early growth through transient mycoheterotrophy. Although both involve intracellular fungal penetration, they evolved independently from AM (Brundrett & Tedersoo, 2018).

Box 2 **Mycorrhizal fungal epigenetics**

Mycorrhizal fungal epigenetics remains a largely unexplored frontier. Few studies have examined histone modifications, and current knowledge mainly derives from DNA methylation profiling in the arbuscular mycorrhizal (AM) fungus *Rhizophagus irregularis* and the ectomycorrhiza (EcM) fungus *Laccaria bicolor*. In *R. irregularis*, 5‐methylcytosine (5mC) levels are high (*c*. 32.5–49.5%), whereas N6‐methyladenine (6mA) is low (*c*. 0.2%) (Chaturvedi *et al*., 2021). Despite its low abundance, 6mA is enriched in genes associated with phosphorus transport, metabolism, and signaling (key symbiotic functions) and is positively associated with transcription. Because plants can transfer mobile small RNAs (sRNA) to fungal partners to direct 5mC‐based gene silencing (Tamiru *et al*., 2018; Huang *et al*., 2019), 6mA may help protect essential fungal genes from host‐induced repression (Chaturvedi *et al*., 2021).

In *L. bicolor*, at least one candidate 6mA methyltransferase gene has been identified (Bewick *et al*., 2019), suggesting a functional 6mA pathway, although its abundance, genomic distribution, and regulatory significance remain unknown. As in other eukaryotes, 5mC is enriched in repeats and transposable elements (TEs), consistent with a conserved genome‐defense role (Feng *et al*., 2010; Zemach *et al*., 2010; Bewick *et al*., 2019). Highly 5mC‐methylated genes show the lowest expression, although no consistent transcriptional association has been found (Bewick *et al*., 2019). The *L. bicolor* epigenome has also been profiled during mycorrhiza formation with poplar, identifying candidate symbiosis‐related genes under epigenetic regulation in both partners (Vigneaud *et al*., 2023).

Distinct mycorrhizal symbiosis types may be regulated by different epigenetic strategies, reflecting variation in plant–fungal intimacy and mutual dependence. For example, intracellular AM fungi may retain 6mA to protect key symbiosis‐related genes from host‐induced sRNA silencing (Chaturvedi *et al*., 2021), whereas intercellularly developing EcM fungi may experience weaker selection on this mechanism. Likewise, AM fungi may require tighter epigenetic control of TEs to maintain genome stability during intracellular colonization.

Box 3 **Core epigenetic mechanisms in plants**

Epigenetic regulation in plants relies on ‘writers’, which add chemical marks, ‘erasers’, which remove them, and ‘readers’, which interpret them. Together, they act across DNA methylation, histone modifications, and noncoding RNAs (Fig. 1).

The main DNA methylation mark 5‐methylcytosine (5mC) occurs in CG, CHG, and CHH sequence contexts (H = A, T or C) (Finnegan *et al*., 1998). *De novo* methylation uses the RNA‐directed DNA methylation (RdDM) pathway, where small RNAs (sRNAs) guide DOMAINS REARRANGED METHYLTRANSFERASE 2 (DRM2) to target loci (Matzke & Mosher, 2014; Xie *et al*., 2025). Symmetrical CG/CHG are maintained by METHYLTRANSFERASE 1 and CHROMOMETHYLASE 3 (CMT3), and asymmetrical CHH by DRM2 (euchromatin) or CMT2 (heterochromatin) (Niederhuth & Schmitz, 2017; Xie *et al*., 2025). Demethylation occurs actively via REPRESSOR OF SILENCING 1 and DEMETER‐LIKE enzymes or passively through replication without maintenance (Xie *et al*., 2025). Although some 5mC persists through meiosis, widespread reprogramming, especially in the male germline, restricts inheritance (Becker *et al*., 2011; Wibowo *et al*., 2016).

Histone modifications regulate chromatin accessibility and recruit regulatory complexes (Kouzarides, 2007). Some histone marks are mitotically stable, while meiotic inheritance is rare (Lämke & Bäurle, 2017). In plant–mycorrhizal interactions, arbuscular mycorrhiza colonization alters histone deposition/removal gene expression (Shu *et al*., 2021). Studies in pathogenic fungi, endophytes, and rhizobial symbioses show histone remodeling during colonization (Chujo & Scott, 2014; Wang *et al*., 2020; Zhang & Tao, 2025), suggesting similar roles in mycorrhizal symbiosis.

Noncoding RNAs integrate these layers: sRNAs direct RdDM to establish *de novo* 5mC (Matzke *et al*., 2015; Zhang *et al*., 2018), while long ncRNAs act as scaffolds for chromatin modifiers (Ariel *et al*., 2015). Importantly, sRNAs can move systemically within plants and direct DNA methylation in distant tissues (e.g. via phloem transport) (Molnar *et al*., 2010).

## II. Epigenetic regulation of mycorrhizal symbioses

**Fig. 2:**
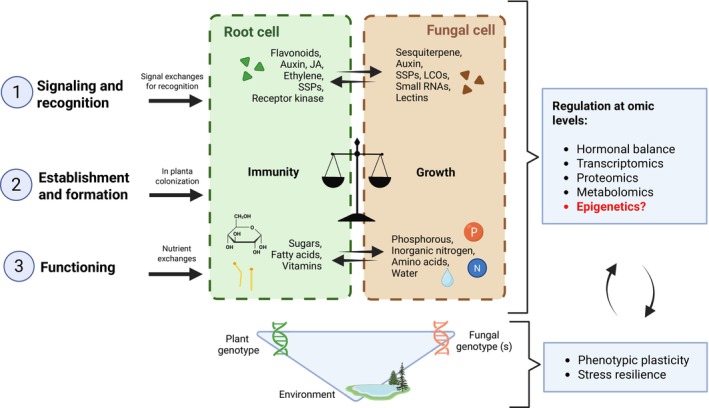
Overview of the signaling, establishment, and functioning phases of mycorrhizal symbiosis and their regulation across molecular and environmental levels. Mycorrhizal development proceeds through three interconnected stages: (1) Signaling and recognition: A bidirectional exchange of signaling molecules initiates recognition between the root and fungal cells. Plant‐derived flavonoids, auxin, jasmonate (JA), ethylene, and small secreted proteins (SSPs) interact with fungal molecules such as sesquiterpenes, auxin, SSPs, lipo‐ and chitooligosaccharides (LCOs/COs), small RNAs, and lectins. Signal perception by receptor kinases in root cells triggers transcriptional reprogramming for symbiotic compatibility. (2) Establishment and formation: Following recognition, fungal *in planta* colonization occurs, coordinated by a balance between plant immunity and fungal growth (illustrated by a scale). This balance is modulated by phytohormones and environmental cues that fine‐tune the accommodation and development of fungal structures within root tissues. (3) Functioning: Mature symbiosis enables nutrient exchanges between partners. Root cells supply sugars, fatty acids, and vitamins, while fungal cells deliver phosphorus (P), inorganic nitrogen (N), amino acids, and water. Regulatory processes at the omic level (hormonal signaling, transcriptomics, proteomics, metabolomics, and potentially epigenetics) modulate each phase and mediate crosstalk between plant and fungal partners. Interactions among plant genotype, fungal genotype, and environment influence the expression of mycorrhiza‐related traits, ultimately shaping phenotypic plasticity and stress resilience. JA, Jasmonate; LCO/CO, Lipo‐/Chitooligosaccharides; SSP, small secreted proteins. This figure was created in BioRender (BioRender.com/y3wn7ah).

Mycorrhizal symbiosis proceeds through three main phases: (1) signaling and recognition, in which plants and fungi exchange molecular signals, enabling hosts to distinguish potential symbionts from pathogens; (2) colonization and establishment, involving local suppression of host defense responses and coordinated remodeling of root cell architecture; and (3) functioning, during which specialized interfaces (e.g. arbuscules in AM symbiosis) mediate reciprocal nutrient exchange (Fig. 2).

Emerging studies indicate that chromatin‐based regulation plays important roles throughout these stages: DNA methylation and histone modifications modulate the transcription of symbiosis‐related genes during early signaling and colonization, whereas sRNAs contribute to transcriptional reprogramming during nutrient exchange (Chaturvedi *et al*., 2021; Wong‐Bajracharya *et al*., 2022). Although direct evidence from fungi remains limited, available data suggest that similar regulatory processes are likely to operate in fungal partners as well, indicating a coordinated epigenetic dialogue between hosts and fungal symbionts (Montanini *et al*., 2014; Wong‐Bajracharya *et al*., 2022).

Despite these advances, the mechanistic understanding of chromatin dynamics in mycorrhizal symbiosis remains incomplete, particularly regarding potential across‐generational effects. Some insight comes instead from plant–pathogen and plant–endophyte interactions, in which epigenetic processes underpin stress response and heritable immune priming (Dowen *et al*., 2012; Secco *et al*., 2015; Liu *et al*., 2024; Ramatsitsi & Manyevere, 2025). Because endophytic fungi colonize plant tissues in largely asymptomatic ways, their parallels with mycorrhizal symbiosis provide a useful comparative framework.

### 1. Mycorrhizal signaling and establishment: hormones and defense regulation

The establishment and functioning of symbiosis require continuous signal exchange that coordinates metabolic and structural adjustments in both partners (Gianinazzi‐Pearson, 1996; Vierheilig, 2004). Symbiosis recognition must suppress immune responses while still allowing systemic defense priming known as mycorrhiza‐induced resistance (Jung *et al*., 2012; Cameron *et al*., 2013). These recognition and immune‐defense processes in mycorrhizal symbiosis are regulated by hormonal crosstalk. In AM symbiosis, salicylic acid (SA) (a defense hormone) transiently peaks early as the plant attempts to reject the fungus, then declines to allow colonization. Meanwhile, jasmonic acid (JA, typically another defense hormone) and abscisic acid (a growth/stress hormone) rise to optimal levels that promote arbuscule development and fungal progression rather than defense activation (Bedini *et al*., 2018; Maury *et al*., 2019). In EcM symbiosis, from what is known for the gray poplar (*Populus tremula × alba*) – *L. bicolor* model, SA accumulates during mid‐stage establishment, while colonized roots show reduced JA sensitivity compared with uncolonized roots, indicating that EcM fungi actively suppress the plant's JA‐mediated defense responses (Basso *et al*., 2020). Thus, both AM and EcM fungi counteract plant defense hormones (early SA suppression in AM, JA signaling suppression in EcM) to create a symbiosis‐permissive environment through convergent strategies.

Epigenetic mechanisms may fine‐tune the symbiosis by modulating signaling cascades and hormonal pathways in both partners (García‐Garrido & Ocampo, 2002; Pozo *et al*., 2015). In gray poplar, experimental manipulation of DNA methylation disrupted hormone‐ and immunity‐related gene expression, impairing EcM root colonization (Vigneaud *et al*., 2023). Fungal partners also contribute the AM fungus *R. irregularis* and the EcM fungus *Pisolithus microcarpus* produce sRNAs that are transferred into plant host cells to promote root colonization (Wong‐Bajracharya *et al*., 2022; Silvestri *et al*., 2025), likely by suppressing host immune responses through alterations in gene expression. Although these studies demonstrate fungus‐to‐plant cross‐kingdom RNA transfer, whether fungal sRNAs induce host chromatin modifications remains unknown.

Comparable mechanisms are well‐documented in plant–pathogen and plant–endophyte fungal interactions, in which fungi manipulate host chromatin through changes in DNA methylation, histone modifications, and sRNA‐mediated gene silencing to suppress immunity and facilitate colonization (Agarwal *et al*., 2020; Panigrahi *et al*., 2021; Sampson *et al*., 2024). Cross‐kingdom RNA interference is also well‐established in pathogenic systems, with sRNAs functioning as effectors that manipulate host defense pathways, and plants reciprocally producing sRNAs that target fungal virulence genes (Weiberg *et al*., 2013; Huang & Jin, 2022). Endophytic fungi can similarly induce host epigenetic reprogramming, including DNA methylation changes that modulate plant gene expression and facilitate asymptomatic colonization; in some cases, these interactions have been linked to enhance abiotic stress tolerance (Sun *et al*., 2012; Woodward *et al*., 2012), although the generality of this benefit remains context‐dependent. These similarities among fungi forming several types of interactions suggest that mycorrhizal fungi operate within a broader continuum of fungal lifestyle strategies that rely on conserved chromatin‐based tools to modulate host immunity.

Another key regulatory target is the host ubiquitinome, the set of proteins regulated by ubiquitination (Inès *et al*., 2025), a post‐translational modification that controls protein stability, localization, and activity, and is central to immune signaling and chromatin remodeling (Kramer *et al*., 2023). A nuclear effector from *R. irregularis* interferes with host H2B mono‐ubiquitination, reshaping ubiquitin‐mediated regulation to suppress defense and promote arbuscule formation (Wang *et al*., 2021; Inès *et al*., 2025). These studies suggest interplay between hormonal signaling, immune regulation, and host–fungal epigenetic control during mycorrhizal establishment, with epigenetic processes acting as active regulators rather than passive by‐products. Nonetheless, direct mechanistic links between chromatin states and colonization efficiency remain largely unexplored.

### 2. Mycorrhiza functioning: mineral nutrient and carbon exchange

Once established, symbiosis shifts from recognition and establishment to functioning, and centers on nutrient exchange, primarily phosphorus (P) and nitrogen (N) against carbon (C) (Bennett & Groten, 2022). AM fungi specialize in inorganic phosphate (Pi) uptake and delivery through arbuscules (Smith *et al*., 2003; Smith & Smith, 2011), whereas EcM fungi dominate N acquisition from inorganic (NH_4_^+^, NO_3_^−^) and organic (i.e. amino acids) sources (Tedersoo & Bahram, 2019). AM fungi also contribute to N uptake, although their role is more variable and often depends on P nutrition (Boussageon *et al*., 2022). In return, plants allocate carbon to fungi, estimated at 1–13% of net primary productivity, depending on the mycorrhizal type (Smith & Read, 2008; Hawkins *et al*., 2023). This bidirectional exchange forms the core of the functioning of mycorrhizal symbiosis and determines to which degree the interaction is mutualistic or parasitic (Bennett & Groten, 2022).

Although direct evidence is lacking for chromatin control of nutrient exchange in mycorrhizas, P and N homeostasis is known to be influenced by epigenetic mechanisms in non‐symbiotic contexts (Li *et al*., 2012; Fan *et al*., 2021). In *Arabidopsis thaliana*, Pi starvation triggers chromatin remodeling via the histone variant H2A.Z and DNA methylation changes, associated with transcriptional changes of Pi‐responsive genes (Smith *et al*., 2003; Yong‐Villalobos *et al*., 2015). In rice (*Oryza sativa*), Pi starvation triggers transcriptional changes of Pi starvation‐induced genes, and then DNA methylation changes, mostly at nearby transposable elements (TEs) (Secco *et al*., 2015). N limitation also induces DNA methylation changes that can even be inherited across generations, enhancing tolerance to low N (Kou *et al*., 2011). In maize (*Zea mays*), N and P deficiencies elicit distinct methylation responses: While N deficiency causes extensive DNA methylation loss, P deficiency has weaker effects (Mager & Ludewig, 2018). Most changes occur in TEs and are linked to transcriptional regulation of adjacent genes. These findings suggest nutrient‐ and species‐specific methylome remodeling and highlight that TE methylation could be a potential regulatory switch during nutrient stress and, by extension, could modulate gene expression during mycorrhizal functioning.

Evidence for ncRNA regulation in mycorrhizal functioning is still lacking, but insights from other biotic interactions further suggest similar mechanisms for chromatin regulation. In tomato (*Solanum lycopersicum*), the transcription factor WRKY75, a key regulator of Pi homeostasis involved in P starvation responses (Devaiah *et al*., 2007), is strongly induced during infection with *Botrytis cinerea*, *Pseudomonas syringae*, and herbivory (Finiti *et al*., 2014; López‐Galiano *et al*., 2018). Its activation involves both a noncanonical microRNA and the deposition of activating H3K4me3 histone marks. The authors further propose that this microRNA might direct DNA methylation via RNA‐directed DNA methylation (RdDM) at WRKY75 nearby genomic regions (López‐Galiano *et al*., 2018). Together, these results illustrate how ncRNAs and chromatin modifications can jointly modulate a transcription factor having dual functions: nutrient homeostasis and defense. Although this has not yet been demonstrated in mycorrhizal systems, these findings illustrate a mechanistic framework that could be tested to determine whether ncRNAs similarly modulate dual‐function regulators in mycorrhizal symbiosis. This would allow plants to optimize nutrient exchange without compromising defense, echoing the strategies observed in other biotic interactions.

Supporting this idea, fungal epigenetics can also influence symbiotic functioning. In the EcM fungus *L. bicolor*, experimental RNA silencing of the nitrate reductase gene using a hairpin transgene leads to localized CG methylation and transcriptional repression at this specific locus, impairing fungal growth on nitrate and preventing symbiosis with Populus unless an organic N source usable by the fungus but not by the plant is supplied (Kemppainen *et al*., 2009). This result suggests that the plant can monitor the nutritional status of its fungal partner and avoid supporting the association when nutrient exchange is unbalanced. In pathogenic and endophytic fungi, epigenetic modifications of virulence or nutrient‐acquisition‐related gene clusters directly influence host defense responses and fungal colonization success (Dean *et al*., 2012; Sun *et al*., 2012; He *et al*., 2020). These examples highlight how epigenetic regulation of fungal nutrient uptake genes can directly affect symbiotic compatibility and functioning, implying that the epigenetic state of key fungal metabolic genes may determine whether mutualism is sustained. Furthermore, they suggest that dynamic epigenetic regulation of key metabolic and virulence genes may represent a conserved mechanism across mutualistic and antagonistic plant–fungal interactions.

Carbon allocation in the form of sugars and lipids represents a key regulatory point in mycorrhizal symbiosis. AM fungi receive lipids synthesized by the host and exported via ATP binding cassette transporters, while sugar efflux is mediated by transporters of the SWEET (Sugars Will Eventually be Exported Transporters) family (Jiang *et al*., 2017; Keymer *et al*., 2017; Salmerón‐Santiago *et al*., 2021; Bell *et al*., 2024). In EcM systems, fungi can modulate the expression of host sugar transporters, such as SWEETc in Poplar, to optimize carbon supply during colonization (Li *et al*., 2024). Similar transcriptional regulation is observed in plant–pathogen interactions, in which pathogens alter transporter expression to access host sugars (Breia *et al*., 2021; Chen *et al*., 2023). These observations highlight that controlling sugar transport is a common mechanism across biotic interactions, while the potential role of epigenetic modifications in fine‐tuning carbon allocation in mycorrhizal symbioses remains an important topic for future research.

Together, these findings highlight the potential for epigenetic mechanisms to mediate mycorrhizal functioning by fine‐tuning nutrient uptake and carbon allocation in both partners. While direct links between chromatin states, transport processes and fungal metabolism during symbiosis are scarce, parallels with plant–pathogen systems suggest conserved epigenetic mechanisms. Importantly, transgenerational immune priming is well‐documented in plant–pathogen systems, in which stress‐induced epigenetic modifications enhance offspring resistance across generations (Luna *et al*., 2012; Herman & Sultan, 2016). This raises the question of whether similar across‐generational epigenetic inheritance exists in mycorrhizal symbioses. Although mycorrhizal interactions have been shown to generate adaptive intergenerational effects on plant performance (Varga *et al*., 2013; Puy *et al*., 2022; Latzel *et al*., 2025), no studies have yet investigated whether mycorrhizal colonization induces heritable epigenetic changes. Addressing this knowledge gap is essential for clarifying the occurrence and extent of epigenetic regulation in mycorrhizal symbiosis, and for understanding its potential biological, ecological and evolutionary consequences.

## III. From within‐ to across‐generational epigenetics: knowledge gaps, challenges, and applications

### 1. Knowledge gaps and open questions

**Fig. 3:**
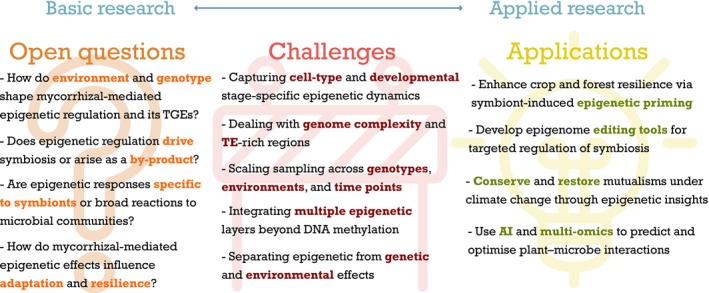
Conceptual framework linking open questions, challenges, and applications in the study of mycorrhizal‐mediated epigenetic regulation. The open questions (left) highlight current knowledge gaps on how environmental factors, plant genotype, and microbial interactions shape transgenerational epigenetic effects (TGEs). These questions feed into the methodological and conceptual challenges (center) that must be addressed to disentangle epigenetic dynamics across cell types, genomes, environments, and timescales. The connecting line from open questions → challenges → applications illustrates how resolving these obstacles is essential for translating basic research into applied research, including crop and forest resilience, targeted epigenome editing, conservation of plant‐fungus mutualisms under climate change, and artificial intelligence (AI)‐driven prediction of plant–microbe interactions.

Despite extensive research on plant–fungus symbioses, the mechanisms by which mycorrhizal fungi and other soil microbes influence host epigenetic pathways remain poorly understood (Fig. 3). Evidence shows that mycorrhizal fungi enhance growth and stress tolerance by modulating plant responses to nutrient limitation, drought, and pathogens (Diagne *et al*., 2020). However, it remains unclear whether these interactions directly shape epigenetic pathways, particularly the mechanisms underlying across‐generational effects and their consistency across plant genotypes, species, and environments. A critical gap is whether mycorrhizal interactions leave reproducible chromatin ‘footprints’ that are both ecologically robust and relevant for the functional outcome of the symbiosis.

A second major open question concerns the causal role of epigenetic regulation in symbiosis. It remains unresolved whether epigenetic regulation actively drives recognition, establishment, and functioning of mycorrhizas or instead represents a downstream consequence of signaling and metabolism. TEs highlight this ambiguity: Their activation can be either repressed or unleashed by epigenetic pathways, and in turn TE activity may provide the regulatory flexibility needed to generate rapid changes in gene expression in response to both beneficial and antagonistic microbes (Anca *et al*., 2014; Vangelisti *et al*., 2019). Clarifying whether epigenetic mechanisms are confined to a restricted set of symbiosis‐related genes or instead reprogram broader regulatory networks affecting plant fitness and microbial cooperation will be essential for linking chromatin‐level regulation to ecological stability and symbiotic efficiency.

A third limitation lies in the narrow focus on mycorrhizal fungi. It remains unclear how specific epigenetic marks are written, maintained, and inherited in mutualistic fungi themselves. Whether mutualistic fungi rely on mechanisms similar to well‐studied pathogens or follow distinct strategies adapted to cooperation is an open question. Other members of the root and soil microbiome, including bacteria, nonmycorrhizal fungi, and archaea, remain largely unexplored despite their potential to modulate plant epigenetic responses (Chen *et al*., 2022; Sizmur & Larionov, 2025; Zou *et al*., 2025). It is not yet known whether plants mount symbiont‐specific epigenetic changes or display broad, nonspecific responses to microbial community composition, nor whether these responses persist across developmental stages or clonal/sexual generations. The lack of integration between microbiome ecology and epigenetic regulation limits our ability to predict how complex microbial communities influence epigenetic memory, plant adaptation, and the recruitment of beneficial partners. Comparative studies across the continuum of plant–microbe interactions, from mutualism (e.g. AM, EcM), through neutral associations (e.g. endophytes), to antagonistic interactions (e.g. pathogens, parasites), are needed to assess whether epigenetic signatures differ and align with contrasting ecological and evolutionary outcomes.

Finally, the long‐term stability and evolutionary significance of mycorrhiza‐induced epigenetic changes remain poorly understood. Although environmentally induced epigenetic modifications can persist for one or a few offspring generations (Sammarco *et al*., 2024, 2025), long‐term inheritance may occur under strong or repeated stress (Herman & Sultan, 2016; López *et al*., 2024). Whether mycorrhizal interactions consistently induce inherited modifications across generations, there are critical developmental windows for epigenetic reprogramming, and how clonal vs sexual reproduction modulates these processes are open questions. Moreover, the extent to which plant and fungal genotype variations influence the strength and persistence of across‐generational effects, and whether trade‐offs exist between short‐term benefits to growth and stress tolerance and long‐term adaptation remains unexplored. The contribution of mycorrhiza‐mediated across‐generational effects to plant adaptation, local adaptation, and even speciation is unknown.

Because mycorrhizal symbioses originated over 450 million years ago (Strullu‐Derrien *et al*., 2018), a central question is whether epigenetic regulation has shaped the recognition, establishment, and functioning of these ancient associations, and whether it underpins their remarkable plasticity and persistence across diverse environments. Specifically, we need to determine whether epigenetic mechanisms contribute to the evolutionary resilience and stability of the symbiosis under past and ongoing environmental change, or whether they mainly provide short‐term regulatory flexibility with limited long‐term inheritance. Clarifying the stability, adaptive value, and evolutionary role of mycorrhiza‐induced epigenetic marks is therefore essential for linking molecular regulation to ecological and evolutionary outcomes.

### 2. Challenges

The explanatory potential of epigenetic modifications, integrating genetic and environmental signals to shape phenotypic variation and contributing to adaptation and evolutionary dynamics, has spurred the rapid adoption of epigenomic approaches in ecological and evolutionary studies (Sow *et al*., 2018; Laine *et al*., 2023). Decreasing sequencing costs and improved workflows for DNA methylation have further accelerated their use in ecological and evolutionary studies. Yet, several methodological and conceptual challenges limit progress in the context of plant–fungus symbioses (Fig. 3).

A first challenge relates to the spatiotemporal dynamics and cell‐type specificity of epigenetic modifications. Unlike genetic analyses, epigenetic profiling is highly dependent on developmental stage and cell identity. In nonmodel plant and fungal species, tissue sampling is often guided by pragmatic rather than biological considerations. While single‐cell, cell‐sorting, and statistical deconvolution methods are state‐of‐the‐art in mammalian epigenomics (Michels *et al*., 2013), their application remains limited in plants and fungi due to the lack of epigenome references across multiple cell types and developmental stages. This is particularly problematic for symbioses, in which only a subset of root cells (e.g. arbuscule‐containing cells or cells surrounded by the Hartig net) directly interact with fungi. Single‐cell epigenomic approaches (Stuart, 2024) could overcome this limitation, but technological and cost constraints remain.

A second challenge emerges from genome architecture. The high genetic complexity and abundance of TEs in plants complicate the interpretation of short‐read bisulfite sequencing data, which struggles with repetitive regions. This technique detects DNA methylation at single‐base resolution by converting unmethylated cytosines to uracils through sodium bisulfite treatment, while methylated cytosines remain unchanged (Chen *et al*., 2018). Repetitive regions rich in TEs cause difficulties in read alignment and accurate methylation calling, which can lead to biases and reduced confidence in methylation profiles. Because TEs can become transcriptionally active during stress or symbiosis, their dynamic epigenetic regulation adds complexity by generating molecular noise that obscures the identification of functional regulatory changes in mycorrhizal systems. Joint genetic–epigenetic approaches such as hairpin sequencing (Füllgrabe *et al*., 2023) could help but remain underexplored in species with non‐CG methylation contexts. Similarly, long‐read sequencing technologies such as Oxford Nanopore and PacBio offer the potential to directly detect DNA methylation marks, including N6‐methyladenine reported in some mycorrhizal fungi (Chaturvedi *et al*., 2021). Although concerns remain about their accuracy in calling methylation states, rapid advances in computational tools have greatly improved their reliability (Fu *et al*., 2025; Liu & Conesa, 2025), making these methods increasingly attractive to study host–microbe systems.

A third challenge concerns sampling scale. Capturing the diversity of epigenetic responses across genotypes, developmental stages, and environments requires large datasets, but whole‐genome bisulfite sequencing remains costly. Reduced representation approaches such as reduced representation bisulfite sequencing have been adapted to plants (Schmidt *et al*., 2017), but their reliance on restriction enzymes leaves many functionally relevant cytosines uncharacterized. To address this, emerging targeted enrichment strategies such as probe‐based capture (SeqCapBis; Lesur *et al*., 2024) and adaptive sampling on Nanopore platforms (Loose *et al*., 2016) offer scalable alternatives with high depth. This shift in scale will help address the challenge of assessing methylation heritability in mycorrhizal symbiosis, especially in pan genomic contexts, as highlighted by a transgenerational study focusing on a single genotype (Panda *et al*., 2023). These methods will be adequate to capture dynamic regulatory shifts from early colonization to mature mycorrhizal symbiosis.

A fourth challenge is the integration of multiple epigenetic layers. DNA methylation is by far the most‐studied mark, yet other modifications, including histone modifications, chromatin accessibility, and sRNAs, act in concert to shape cell‐specific regulatory networks. Multi‐omic tools such as single‐molecule chromatin fiber sequencing (Fiber‐seq) (Stergachis *et al*., 2020) or spatial single‐cell omics (Dezem *et al*., 2024) would provide simultaneous profiling of genetic variation, DNA methylation, nucleosome occupancy, and chromatin accessibility, and would illuminate the fine‐scale cellular interactions at plant–fungus interfaces, as recently applied in flowering plants (Bubb *et al*., 2025). Applying such integrative approaches for arbuscule‐containing cells or EcM root tips would provide unprecedented insights into how plant and fungal chromatin states interact.

Finally, disentangling epigenetic contributions from genetic background effects remains a persistent challenge in multipartner systems, such as plant–fungal symbioses. Variability across plant genotypes, fungal strains, and soil environments hinders reproducibility and generalization. Large and standardized reference datasets across species and environments are needed but are difficult to construct. Machine learning and artificial intelligence (AI) frameworks offer tools to model regulatory networks and to predict phenotypes from multi‐omic features (Bai *et al*., 2024). In particular, deep learning approaches show potential to uncover epigenetic memory and regulatory principles by integrating transcriptomic, epigenomic, and chromatin data (Zhang *et al*., 2023; Dobránszki *et al*., 2025). Frameworks developed in well‐characterized organisms (Ballard *et al*., 2024; Tahir *et al*., 2024) may accelerate their application to plants and, by extension, to plant–fungus symbioses.

Together, these challenges highlight that although technological advances are rapidly expanding the scope of plant and fungal epigenomics, the study of epigenetic regulation in mycorrhizal systems is still constrained by limitations in resolution, sampling design, and integrative analysis. Overcoming these hurdles will be essential to move from correlative observations toward causal understanding, ultimately linking epigenetic modifications with the ecological and evolutionary dynamics of plant–fungus symbioses.

### 3. Toward applications

Epigenetics provides a promising framework for advancing both fundamental and applied research on plant–fungus interactions. However, current understanding remains very limited, and potential applications are speculative. A key unresolved question is the role of epigenetic regulation within the hierarchy of factors controlling plant–mycorrhizal interactions. While genetics, physiology, and environment are major drivers, epigenetic mechanisms offer a flexible and potentially heritable layer of control that may fine‐tune symbiotic efficiency. Future work should clarify whether epigenetic changes act as causes, consequences, or integrators of plant–fungus communication before practical use can be envisioned. Below, we outline several potential yet speculative applications that could emerge as knowledge advances (Fig. 3).

#### Agriculture and forestry

Epigenetic insights could improve crop and tree resilience to abiotic and biotic stress by identifying fungal symbionts that induce stable, beneficial epigenetic modifications in their hosts while displaying high nutrient‐exchange efficiency or biocontrol capacity. For instance, fungal strains that consistently modulate DNA methylation or histone modification states in roots (enhance nutrient exchange and acquisition, drought tolerance, and pathogen resistance) could transmit these benefits across multiple generations. In seed and cutting propagation systems, pre‐inoculating parent plants with fungal strains known to imprint heritable epigenetic modifications may increase the likelihood that progeny will associate with beneficial isolates tailored for optimal growth and stress resilience. These strategies could be particularly relevant for long‐lived and perennial species such as trees, in which epigenetic mechanisms could sustain adaptive responses over decades and support the transmission of stress‐induced states to progeny, thereby contributing to long‐term resilience.

Intra‐specific fungal diversity significantly influences plant performance, which can be partly linked to intrinsic fungal traits such as nutrient trading efficiency and biocontrol capacities (Munkvold *et al*., 2004; Antunes *et al*., 2011; Mensah *et al*., 2015). However, part of the functional variability related to fungal identity could also be due to epigenetic regulation. Thus, combining screening for both epigenetic impacts and key fungal traits may advance improvements in crop and tree performance.

A fundamental challenge is to disentangle the relative contributions of host‐ vs symbiont‐derived epigenetic regulation. If stable modifications are predominantly plant‐controlled, breeding strategies can prioritize genotypes with higher epigenetic responsiveness to symbiosis. Conversely, if the fungal partner exerts stronger epigenetic control, screening for fungal strains with predictable and beneficial epigenetic effects may represent a new breeding paradigm. Importantly, assessing whether these modifications target only symbiosis‐related genes or extend to broader functional and stress‐related pathways will determine whether epigenetic‐assisted breeding can be generalized or must remain context‐dependent.

#### Biotechnology and synthetic biology

Epigenome‐editing technologies, such as CRISPR/dCas9‐based DNA methylation or histone modification tools, open new possibilities for fine‐tuning plant–microbe interactions by directly manipulating key regulatory marks. Such interventions could be transient (priming plants before field deployment) or stable (heritable across generations), depending on the application. Equally important is clarifying the interplay between DNA methylation, histone modifications, and sRNAs. It remains unclear whether they act independently or synergistically in controlling symbiotic pathways. Controlled exposure to environmental cues, such as mild drought or nutrient shifts, may also be used to precondition epigenetic states in plants and fungi, effectively ‘training’ the symbiosis for resilience under fluctuating field conditions.

#### Ecosystem management and conservation

Epigenetic perspectives can inform strategies to preserve and restore beneficial symbioses in ecosystems under climate change. For example, identifying how temperature extremes, altered precipitation or soil degradation modulate the epigenetic regulation of plant–fungus interactions could help predict the stability of mutualisms under future scenarios. Conservation programs could incorporate symbiont selection or management practices that maintain epigenetic resilience, thereby supporting plant establishment, nutrient cycling, and ecosystem functioning.

#### Artificial intelligence (AI) and predictive modeling

Advances in AI and machine learning offer powerful tools to integrate multilayered datasets, including genomic, epigenomic, transcriptomic, metabolomic, and phenotypic profiles, to predict which host‐symbiont pairings yield the most beneficial epigenetic states. Beyond predicting plant–fungus combinations, AI models may identify specific environmental cues that trigger beneficial epigenetic priming, enabling the preconditioning of plants or microbes before field introduction. Such predictive frameworks could accelerate the design of tailored plant–symbiont systems optimized for agricultural productivity or ecological resilience, while helping to clarify the hierarchical role of epigenetics relative to other regulatory factors. Their utility will depend on experimental validation to avoid overfitting and ensure transferability.

In summary, epigenetics holds the potential to become both a diagnostic tool and a management lever for mycorrhizal interactions, bridging fundamental knowledge with real‐world applications. However, assessing its position within the broad hierarchy of genetic, physiological, and environmental parameters remains essential for ensuring its effective use in agriculture, biotechnology, and conservation.

## IV. Conclusions

Although direct evidence remains limited, current studies suggest that epigenetic mechanisms may influence both early stages of mycorrhizal interactions, including signaling, immune modulation, and hormonal balance, as well as the long‐term maintenance of symbiosis by modulating mineral nutrients and carbon exchange. Epigenetic regulation has the unique ability to link rapid physiological responses with potentially heritable modifications that may persist across generations, raising the possibility that such mechanisms operate in mycorrhizal interactions. However, direct empirical evidence for mycorrhiza‐induced epigenetic inheritance remains scarce, and key questions persist regarding the stability, persistence, and adaptive significance of these epigenetic marks across reproductive modes and environmental conditions. Resolving these issues requires integrating fungal epigenetic data with plant‐focused studies. Progress will depend on experiments combining diverse plant and fungal genotypes under contrasting environments, enabling a more complete understanding of how epigenetic memory influences ecological dynamics and evolutionary potential.

Looking forward, the field will benefit from long‐term, multigenerational studies coupled with integrative multi‐omics. Recent advances in single‐cell epigenomics, 3D genome architecture mapping, and computational modeling provide the necessary tools, but their application to plant–fungus systems remains limited. Developing approaches that scale from cellular interactions to population‐level outcomes will be essential to link molecular marks with ecological function and evolutionary trajectories.

From an applied perspective, the potential of mycorrhizal epigenetic priming remains to be tested more broadly in crops, as current evidence suggests that mycorrhizal colonization can trigger systemic and stress‐related epigenetic modifications that may improve plant resilience (Cicatelli *et al*., 2014; Varga & Soulsbury, 2019). Such approaches may reduce fertilizer dependence and enhance tolerance to environmental stress, contributing to climate‐resilient agriculture. More broadly, incorporating fungal epigenetics and cross‐kingdom signaling into this framework will expand opportunities for sustainable crop management and ecosystem restoration.

In conclusion, while most research to date has focused on plant responses, deeper integration of fungal epigenetics and soil microbial diversity will be critical. Understanding how epigenetic information is established, transmitted, and maintained across kingdoms will help explain the long‐term stability of symbioses. Ultimately, positioning epigenetics at the interface of molecular regulation, ecological dynamics, and evolutionary resilience will open new opportunities for both fundamental insight and applied innovation.

## Competing interests

None declared.

## Disclaimer

The New Phytologist Foundation remains neutral with regard to jurisdictional claims in maps and in any institutional affiliations.
